# Green tea extracts ameliorate high-fat diet–induced muscle atrophy in senescence-accelerated mouse prone-8 mice

**DOI:** 10.1371/journal.pone.0195753

**Published:** 2018-04-09

**Authors:** Shintaro Onishi, Mayu Ishino, Hidefumi Kitazawa, Ai Yoto, Yuki Shimba, Yusuke Mochizuki, Keiko Unno, Shinichi Meguro, Ichiro Tokimitsu, Shinji Miura

**Affiliations:** 1 Industry–Academia Collaboration Research Laboratory of the University of Shizuoka and Kao Corporation, University of Shizuoka, Shizuoka, Japan; 2 Laboratory of Nutritional Biochemistry, Graduate School of Nutritional and Environmental Sciences, University of Shizuoka, Shizuoka, Japan; 3 Department of Neurophysiology, School of Pharmaceutical Sciences, University of Shizuoka, Shizuoka, Japan; East Tennessee State University, UNITED STATES

## Abstract

Muscle atrophy (loss of skeletal muscle mass) causes progressive deterioration of skeletal function. Recently, excessive intake of fats was suggested to induce insulin resistance, followed by muscle atrophy. Green tea extracts (GTEs), which contain polyphenols such as epigallocatechin gallate, have beneficial effects on obesity, hyperglycemia, and insulin resistance, but their effects against muscle atrophy are still unclear. Here, we found that GTEs prevented high-fat (HF) diet–induced muscle weight loss in senescence-accelerated mouse prone-8 (SAMP8), a murine model of senescence. SAMP8 mice were fed a control diet, an HF diet, or HF with 0.5% GTEs (HFGT) diet for 4 months. The HF diet induced muscle weight loss with aging (measured as quadriceps muscle weight), whereas GTEs prevented this loss. In HF diet–fed mice, blood glucose and plasma insulin concentrations increased in comparison with the control group, and these mice had insulin resistance as determined by homeostasis model assessment of insulin resistance (HOMA-IR). In these mice, serum concentrations of leukocyte cell–derived chemotaxin 2 (LECT2), which is known to induce insulin resistance in skeletal muscle, were elevated, and insulin signaling in muscle, as determined by the phosphorylation levels of Akt and p70 S6 kinases, tended to be decreased. In HFGT diet–fed mice, these signs of insulin resistance and elevation of serum LECT2 were not observed. Although our study did not directly show the effect of serum LECT2 on muscle weight, insulin resistance examined using HOMA-IR indicated an intervention effect of serum LECT2 on muscle weight, as revealed by partial correlation analysis. Accordingly, GTEs might have beneficial effects on age-related and HF diet–induced muscle weight loss, which correlates with insulin resistance and is accompanied by a change in serum LECT2.

## Introduction

Muscle atrophy—loss of muscle mass—is widely recognized as a risk factor for physical disability and poor quality of life in old age [1–3]. Maintenance of appropriate muscle mass or induction of muscle hypertrophy is achieved by exercise, resistance training, and sufficient nutrient intake [4–7], but muscle atrophy occurs easily with aging and senescence [8–10].

In animal studies, muscle atrophy is typically evaluated by assessing a decline in muscle mass, strength, and fiber type [11, 12]. Hamrick et al. developed a useful model for evaluating muscle atrophy in C57BL/6 mice and clearly showed the loss of hindlimb muscle mass from the age of 18 months [11]. However, their experimental period was too long for investigating the mechanism of muscle atrophy and testing the therapeutic potential of drugs and food ingredients.

Senescence-accelerated mouse (SAM) prone-8 (SAMP8) and senescence-resistant inbred strain 1 (SAMR1) are regarded as appropriate models of muscle aging [12–15]. In SAMP8 mice, Guo et al. revealed that gastrocnemius muscle mass peaked at 7 months and functional and structural decline was observed at 8 months [12].

Muscle mass is ordinarily regulated by the balance of protein synthesis and degradation via insulin signaling [16]. Animal studies suggest that aging-related insulin resistance accompanied by mitochondrial dysfunction and reduction of glucose uptake might suppress protein synthesis [17]. Also, obesity and diabetes are suggested risk factors of muscle atrophy owing to the induction of insulin resistance [18]. Excessive intake of fats is well known to induce obesity and insulin resistance, suppressing signaling upstream of protein synthesis in skeletal muscle [19], but it has not been clarified whether excessive intake of fats exacerbates aging-related insulin resistance and muscle atrophy in SAMP8 mice.

Green tea extracts (GTEs), including highly concentrated polyphenols such as epigallocatechin gallate (EGCg), have been extensively evaluated for their effects on obesity, type II diabetes, hyperglycemia, and insulin resistance in humans and other animals [17, 20–25]. Liu et al. found that intake of food containing 0.3% EGCg for 12 weeks ameliorates aging-related muscle insulin resistance in SAMP8 mice [17], but the effects of GTEs against high-fat (HF) diet–induced muscle atrophy are still unclear.

Leukocyte cell–derived chemotaxin 2 (LECT2) is one of the hepatokines, which have systemic adverse effects; its serum concentrations are elevated in obese and diabetic patients [26, 27]. Lan et al. revealed that serum LECT2 concentrations are elevated by an HF diet in mice and that LECT2-knockout mice have improved insulin sensitivity in skeletal muscle [28]. Although these results suggest that increased concentration of LECT2 reduces insulin sensitivity in skeletal muscle in HF diet–fed mice, it is still unclear whether LECT2 affect muscle atrophy. Also, no reports are available on the effects of GTEs on serum LECT2 concentrations.

In this study, we examined changes in muscle weight (used as an index of muscle mass) during aging in SAMR1 and SAMP8 mice. Then, we compared the changes in muscle mass in control (Cont), HF, and HF with GTEs (HFGT) diet groups to determine the effects of an HF diet and GTEs on muscle atrophy. Progression of insulin resistance and serum concentration of LECT2 were determined to be the likely mechanisms of HF diet–induced loss of muscle mass and the effects of GTEs against HF diet induced–insulin resistance and muscle atrophy.

## Materials and methods

### Ethics statement

Mice were cared for in accordance with the National Institutes of Health Guide for the Care and Use of Laboratory Animals and our institutional guidelines. All animal experiments were conducted with the approval of the Institutional Animal Care and Use Committee of the University of Shizuoka (approval number: 136068). All surgery was performed under anesthesia used 2.5% isoflurane (Wako Pure Chemical Industries, Ltd., Osaka, Japan), and all efforts were made to minimize suffering.

### Animals and diet preparation

Male SAMP8 and SAMR1 mice (1 month old) were obtained from Japan SLC, Inc. (Hamamatsu, Japan). Mice were housed under a 12 h light/dark cycle and allowed free access to CE-2 diet (SLC, Inc.) and water for 3 weeks (pre-breeding period). SAMP8 mice with similar body weights were allocated to three diet groups: Cont, HF, and HFGT. Cont mice were fed a control diet containing 5% corn oil, 20% casein, 4% cellulose, 3.5% minerals (AIN-76), and 1% vitamins (AIN-76). For HF and HFGT mice, the diet was supplemented with 5% lard, 20% corn oil (25% in total), and 13% sucrose. The diet fed to HFGT mice also contained 0.5% GTEs. Total volumes of the diets were adjusted with potato starch (Table 1). SAMR1 mice were fed a Cont diet during the experiment. All mice were fed the Cont diet for 1 week, and then their respective diets were fed for 4 months from 2 months of age (young, 2M) to 6 months of age (adult, 6M). Diet intake was measured three times per week during the experimental period. Calorie-based average daily amount of food intake per mouse (kcal/mouse/day) did not differ significantly among the groups and was 17.00 (SAMR1; Cont), 17.06 (SAMP8; Cont), 19.06 (SAMP8; HF), and 18.13 (SAMP8; HFGT).

**Table 1:** Composition of experimental diets fed to mice.

	Cont	HF	HFGT
Lard	0	5	5
Corn oil	5	25	25
Potato starch	66.5	28.5	28
Sucrose^a^	0	13	13
Casein	20	20	20
Cellulose	4	4	4
Vitamins (AIN-76)	3.5	3.5	3.5
Minerals (AIN-76)	1	1	1
Green tea extracts (GTEs)^b^	0	0	0.5
Energy^c^	%		
Protein	20.5	15.7	15.8
Fat	11.3	51.7	51.9
Carbohydrate	68.2	32.6	32.3

Cont, control diet; HF, high-fat diet; HFGT, HF with 0.5% GTEs diet; diet compositions are indicated in % (w/w).

^a^ Obtained from Wako Pure Chemical Industries, Ltd. (Osaka, Japan)

^b^ Obtained from Mitsui Norin Co., Ltd. (Tokyo, Japan)

^c^ Percent of kcal of each macronutrient

Other ingredients were obtained from Oriental Yeast Co., Ltd. (Tokyo, Japan).

After 5 h of fasting, 8 mice per group at 2M (SAMR1-Cont and SAMP8-Cont) and 16 mice per group at 6M (SAMR1-Cont and SAMP8-Cont, HF, and HFGT) were anesthetized with 2.5% isoflurane. Once anesthesia was confirmed by the loss of pedal reflexes, the abdominal cavity was opened, blood samples were collected from the abdominal vena cava, and the mouse was sacrificed. Blood glucose concentrations were measured using ACCU-CHEK Aviva (Roche Diagnostics, Mannheim, Germany). Immediately after sacrifice, the left side of the quadriceps muscle (as representative skeletal muscle) was dissected and weighted; serum and plasma were obtained from the blood sample and stored at −80°C until analysis.

### Green tea catechin composition and caffeine content

GTEs were purchased from Mitsui Norin Co., Ltd. (Tokyo, Japan). The composition of green tea catechins was determined by high-performance liquid chromatography [29]. Catechin composition of GTEs was shown in Table 2.

**Table 2:** Catechin composition of GTEs.

EGCg, epigallocatechin gallate	71.68
ECg, epicatechin gallate	16.15
GCg, gallocatechin gallate	6.25
EGC, epigallocatechin	2.76
EC, epicatechin	1.24
Cg, catechin gallate	0.77
GC, gallocatechin	0.65
C, catechin	0.24
Others	0.26
Total	100(%)

GTE purity was 77.58%

Caffeine content was 0.20% of total GTEs

The theoretical final content of EGCg in the HFGT diet was 0.28% (a product of multiplication of 0.5% GTEs, 77.58% GTE purity, and 71.68% EGCg composition in total diet). Average daily food intake per mouse in the HFGT group was 3.49 g, and the calculated average daily amounts of total catechins and EGCg intake per mouse were 13.5 and 9.7 mg, respectively (373 and 269 mg/kg-body weight). This dose of EGCg and duration (4 months) were similar to those reported to ameliorate skeletal muscle insulin resistance and fatty liver in SAMP8 mice after 12 weeks of EGCg supplementation [17] and to efficiently inhibit obesity, metabolic syndrome, and fatty liver disease in HF-fed C57BL/6J mice after 16 weeks of EGCg supplementation [25].

### Enzyme-linked immuno-sorbent assay

Plasma insulin concentrations were measured using a Morinaga Mouse Insulin ELISA Kit (Morinaga Institute of Biological Science, Inc., Yokohama, Japan). Serum LECT2 concentrations were measured with an Ab-Match Assembly Mouse LECT2 Kit and Ab-Match Universal Kit (Medical and Biological Laboratories Co., Ltd., Nagoya, Japan). All procedures followed the manufacturers’ protocols. Homeostasis model assessment of insulin resistance (HOMA-IR), an index of insulin resistance, was determined as HOMA-IR = fasting insulin (μU/mL) × fasting blood glucose (mg/dL) / 405. For human insulin, 1 ng/mL = 26 μU/mL.

### Western blot analysis

Western blotting was performed according to the method of Chen et al. [30] with minor modifications. Muscle tissues were obtained as described in the ‘Animals and diet preparation’ section. Frozen muscles were crushed and lysed with RIPA buffer (Wako Pure Chemical Industries) containing cOmplete Protease Inhibitor cocktail (Roche Diagnostics) and a PhosSTOP phosphatase inhibitor (Roche Diagnostics) by following the manufacturer’s protocol. The homogenates were centrifuged, and the supernatants were collected and boiled for 5 min with 50 mM DL-dithiothreitol (Sigma-Aldrich, St Louis, MO) in Laemmli Sample Buffer (Bio-Rad Laboratories, Inc., Hercules, CA). Each sample (10 μg total protein) was subjected to SDS-PAGE through a 4% to 15% gradient gel (Bio-Rad Laboratories, Inc). Proteins were then transferred to polyvinylidene fluoride membranes and incubated for 3 h with PVDF Blocking Reagent for Can Get Signal (TOYOBO Co., Ltd., Osaka, Japan). The membranes were then incubated overnight with primary antibodies diluted 1:1000 in Can Get Signal Solution 1 (TOYOBO Co., Ltd.), followed by incubation for 1 h with anti-rabbit IgG horseradish peroxidase–linked secondary antibody (#7074) or anti-mouse IgG horseradish peroxidase–linked secondary antibody (#7076) (Cell Signaling Technology, Danvers, MA) diluted 1:2000 in Can Get Signal Solution 2 (TOYOBO Co., Ltd.). Signals were detected using the ECL Prime Western Blotting Detection System (GE Healthcare Japan, Tokyo, Japan) and visualized with a luminescence imager (Ez-capture II, ATTO Co., Tokyo, Japan). Primary antibodies against Akt (also known as protein kinase B) (#9272), phospho-Akt (Ser473) (#4058), p70 S6 kinase (S6K) (#9202), phospho-S6K (Thr389) (#9205), ubiquitin (#3936), Forkhead box protein O1 (FoxO1) (#2880), Forkhead box protein O4 (FoxO4) (#9472), and phosphorylation of FoxO1 (Thr24) and FoxO4 (Thr28) (#9461) were purchased from Cell Signaling Technology. Primary antibodies against muscle RING-finger protein-1 (MuRF1) (Ab172479) and F-Box protein 32 (Fbx32) (#168372) were purchased from Abcam plc, (Cambridge, UK).

### Statistical analysis

**Fig 1:**
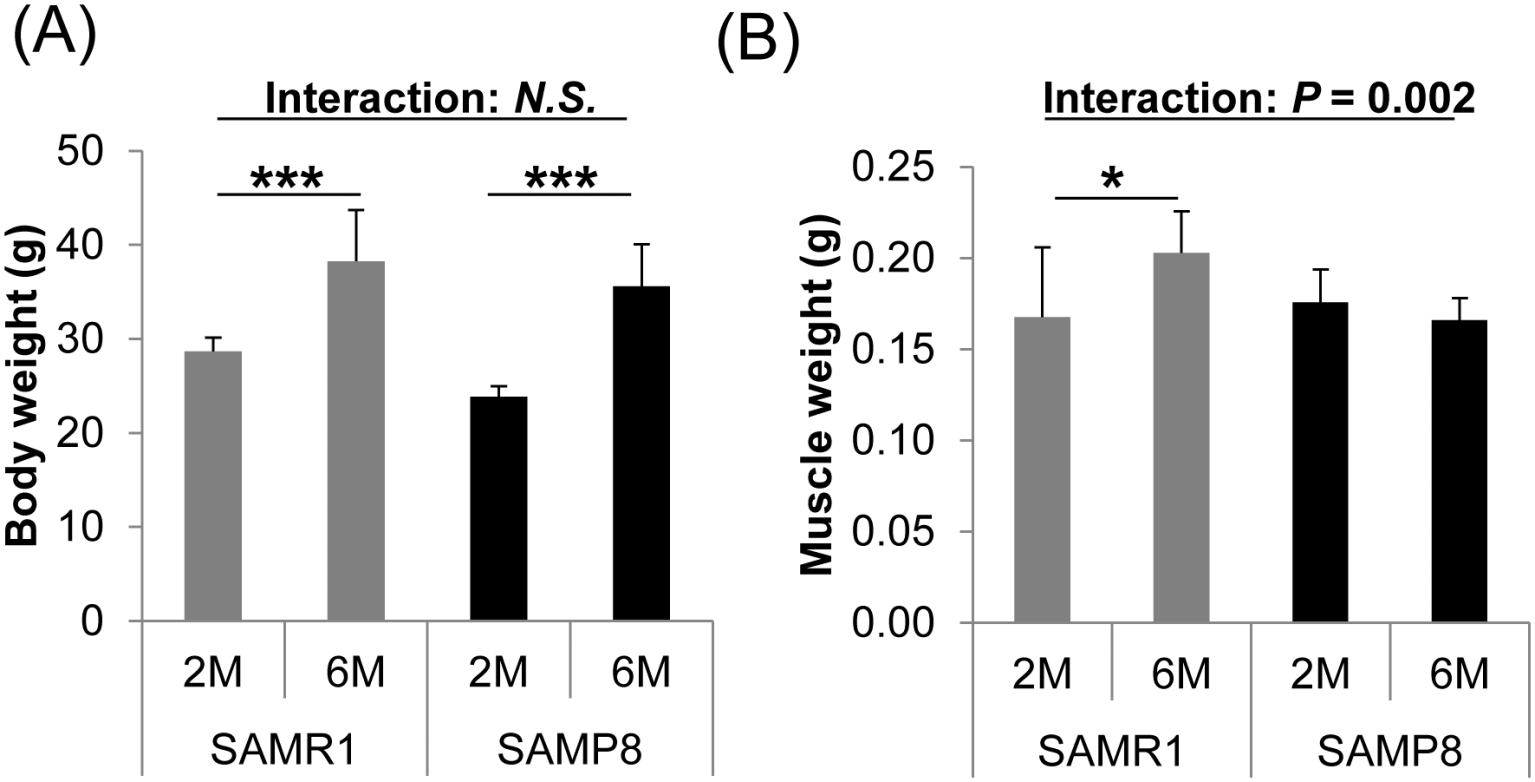
Body weight and skeletal muscle weight in aging SAMR1 and SAMP8 mice. Body weight (A) and skeletal muscle weight (B) were measured in young (2M) and adult (6M) mice fed a Cont diet. Skeletal muscle weight increased in SAMR1 mice concomitantly with body weight gain, but did not increase in SAMP8 mice. Data are means ± S.D. (8 to 16 mice per group). Statistical significance of the interaction between mouse strain (SAMR1 and SAMP8) and age (2M and 6M) was determined by two-way factorial ANOVA without replication, and Student’s *t*-test was used for comparison between the two age groups. *, *P* < 0.05; ***, *P* < 0.001.

**Fig 2:**
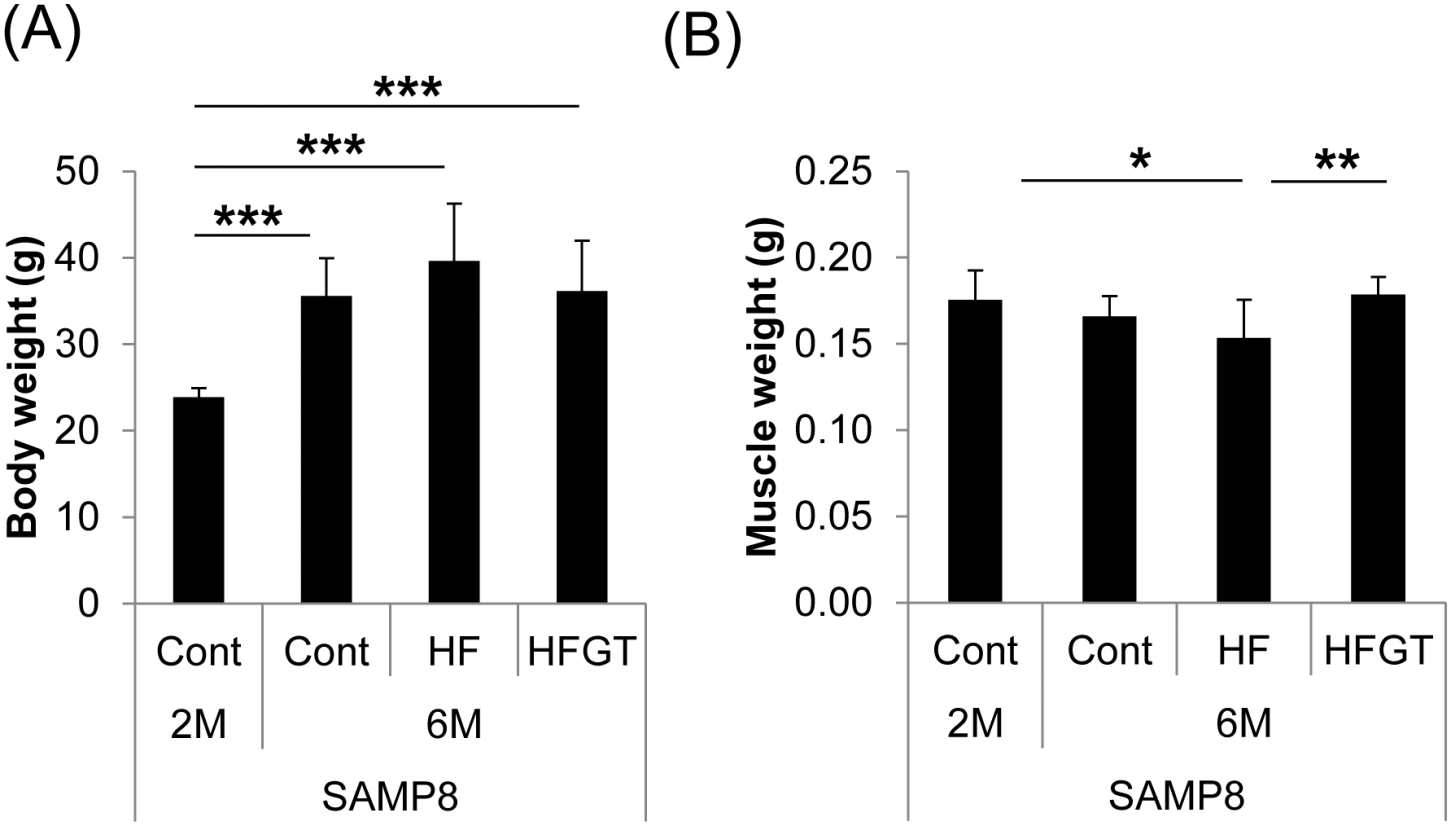
Body weight and skeletal muscle weight in SAMP8 mice on different diets. Body weight (A); skeletal muscle weight (B). Skeletal muscle loss with aging was exacerbated by an HF diet (2M Cont vs. 6M HF; *P* = 0.016) but was significantly prevented by GTEs in the HFGT group (HF vs. HFGT; *P* = 0.002). Data are means ± S.D. (8 to 16 mice per group). One-way ANOVA followed by Tukey’s post-hoc test was used for comparison among groups. *, *P* < 0.05; **, *P* < 0.01; ***, *P* < 0.001.

**Fig 3:**
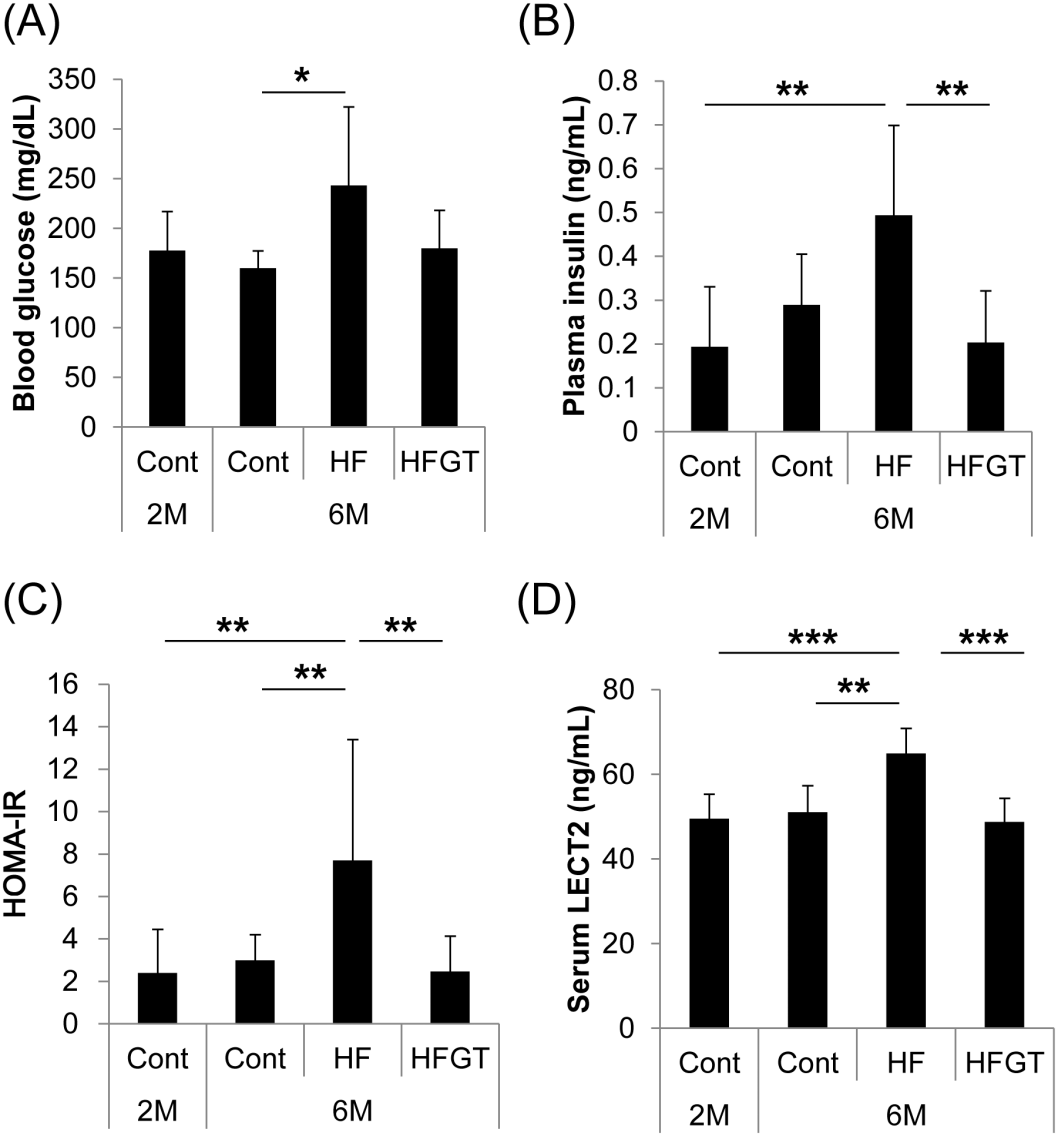
Blood glucose, plasma insulin, HOMA-IR, and serum LECT2 concentrations in SAMP8 mice. Blood glucose levels were analyzed at dissection after 5 h of fasting by ACCU-CHEK Aviva described in Materials and Methods (A). Plasma insulin (B) and serum LECT2 (D) concentrations were analyzed after serum and plasma sample collection by enzyme-linked immuno-sorbent assay. HOMA-IR was calculated as described in the Materials and Methods (C). Data are means ± S.D. One-way ANOVA followed by Tukey’s post-hoc test was used for comparison among groups. *, *P* < 0.05; **, *P* < 0.01; ***, *P* < 0.001.

**Fig 4:**
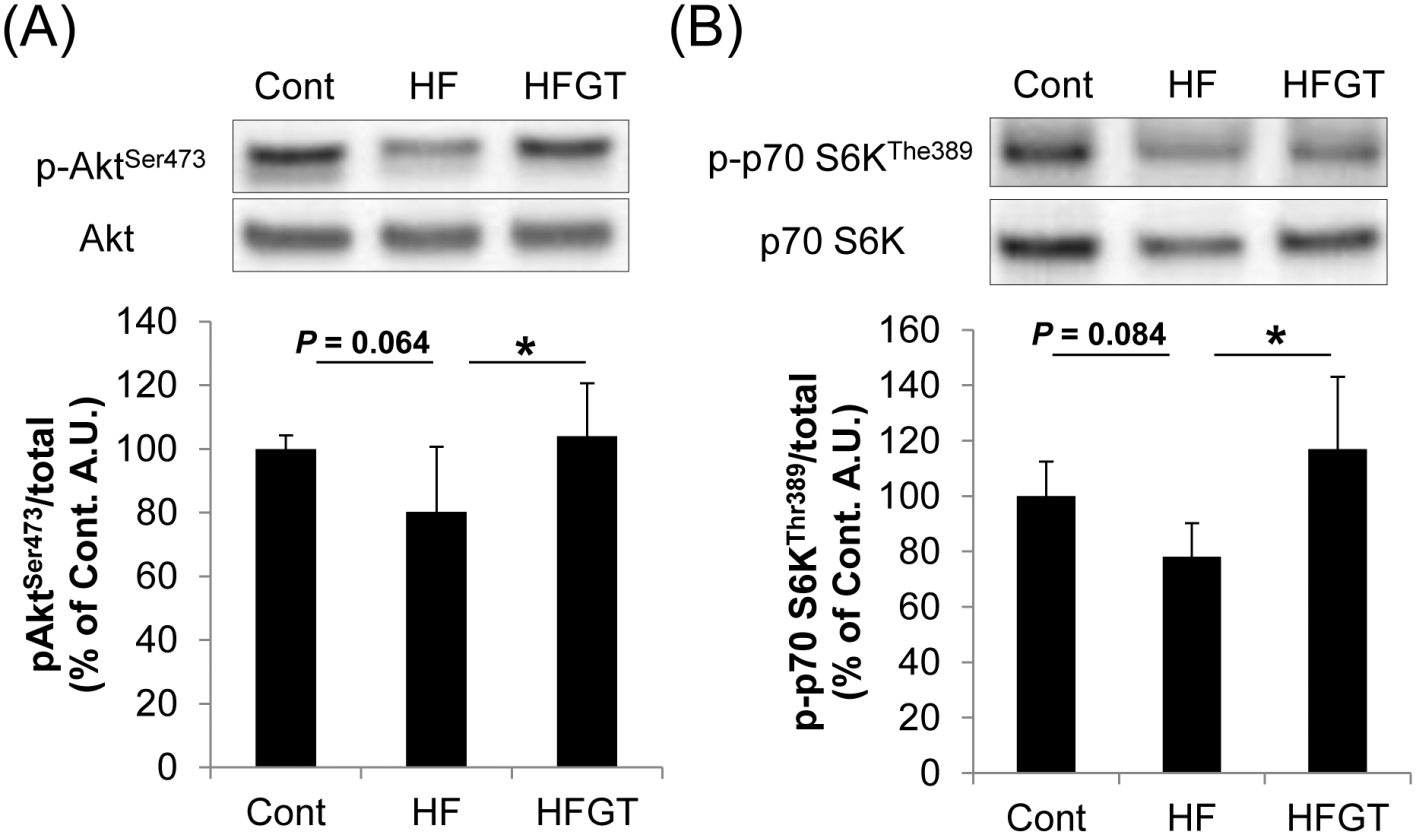
Phosphorylation of kinases involved in insulin signaling in skeletal muscle of adult (6M) SAMP8 mice. Representative western blot images (upper panels, all images provided in S1 Appendix) and quantification of phosphorylation (lower graphs) are shown for Akt (A) and S6K (B). Data are means ± S.D. One-way ANOVA followed by Tukey’s post-hoc test was used for comparison among groups. *, *P* < 0.05.

Data are expressed as means ± S.D. All data were analyzed using IBM SPSS Statistics Version 24 (IBM Corp., Armonk, NY). Statistical significance of the differences between mouse strains (SAMR1 and SAMP8) and ages (2M and 6M) in Fig 1 was determined by two-way factorial ANOVA without replication, followed by Student’s *t*-test. Statistical significance of the differences among groups in Figs 2–4 was determined by one-way ANOVA followed by Tukey’s post-hoc test. Correlation coefficients for muscle weight, HOMA-IR, and serum LECT2 shown in Table 3 were obtained using bivariate Pearson correlation. Partial correlation coefficients shown in Table 4 were obtained using partial correlation analysis. Values of *P* < 0.05 were considered significant.

**Table 3:** Matrix of bivariate correlations among muscle weight, HOMA-IR, and serum LECT2.

Variable		HOMA-IR	Serum LECT2	Muscle weight
HOMA-IR	Pearson correlation	1	0.437**	−0.455**
	*P*-value		0.007	0.005
Serum LECT2	Pearson correlation		1	−0.333*
	*P*-value			0.033
Muscle weight	Pearson correlation			1
	*P*-value			

*Correlation is significant at the P < 0.05 level (1-tailed)

**Correlation is significant at the P < 0.01 level (1-tailed)

**Table 4:** Partial correlations among muscle weight, HOMA-IR, and serum LECT2.

Control variable	Variable	Coefficient	*P*-value
Serum LECT2	HOMA-IR & Muscle weight	-0.365*	0.024
HOMA-IR	Serum LECT2 & Muscle weight	-0.168	0.188
Muscle weight	HOMA-IR & Serum LECT2	0.340*	0.033

*Correlation is significant at the *P* < 0.05 level (1-tailed)

## Results

### Effects of aging on body composition

Body weight increased from the age of 2M to 6M in SAMR1 (normally aging mice) and SAMP8 (senescence-accelerated mice) (Fig 1A). Skeletal muscle weight gain (an index of muscle mass) was observed only in SAMR1 mice (Fig 1B). The changes in muscle mass with aging were significantly different between SAMR1 and SAMP8 mice (interaction between mouse strain (SAMR1 and SAMP8) and age (2M and 6M) was significant; *P* = 0.002).

### Effects of GTEs on HF diet–induced loss of muscle mass in SAMP8 mice

We next determined the effects of diet on the loss of muscle weight with aging in SAMP8 mice. Muscle weight with aging (0.176 ± 0.018 g at 2M to 0.170 ± 0.116 g at 6M) in the Cont diet group was exacerbated in the HF diet group (0.149 ± 0.216 g, *P* = 0.016; 2M Cont vs. 6M HF) despite body weight gain. The reduction of muscle mass weight by the HF diet was significantly prevented by GTEs in the HFGT group (0.182 ± 0.126 g, *P* = 0.002 HF vs. HFGT) (Fig 2).

### Effects of GTEs on insulin resistance and serum LECT2 concentrations in SAMP8 mice

We investigated the state of insulin resistance and serum LECT2 concentrations in SAMP8 mice. Blood glucose, plasma insulin concentrations, and HOMA-IR were higher in the HF group than in the other groups (Fig 3A–3C). Serum LECT2 concentrations were also significantly elevated in the HF group (Fig 3D). Intake of GTEs prevented HF diet–induced elevation of HOMA-IR and an increase in serum LECT2.

### Phosphorylation of kinases involved in insulin signaling in skeletal muscle

To determine changes in insulin sensitivity caused by diet, we examined the phosphorylation status of Akt and S6K, which functions downstream of Akt and is important for regulation of protein synthesis [16]. In the HF group, phosphorylation of both kinases tended to be lower than in the Cont group (Fig 4). The HFGT group had significantly higher Akt and S6K phosphorylation than the HF group (Fig 4). These results suggest that GTEs counteract HF diet–induced reduction in insulin sensitivity in skeletal muscle.

### Intervention effect of HOMA-IR on muscle weight and serum LECT2 concentrations

In SAMP8 mice, bivariate Pearson correlations indicated that HOMA-IR was significantly correlated with serum LECT2 concentration (*r* = 0.437, *P* < 0.01) and skeletal muscle weight (*r* = −0.455, *P* < 0.01), and serum LECT2 was significantly correlated with muscle weight (*r* = −0.333, *P* < 0.05) (Table 3). Partial correlations were found between HOMA-IR and muscle weight (*r* = −0.365, *P* < 0.05) and between HOMA-IR and serum LECT2 (*r* = 0.340, *P* < 0.05), but not between serum LECT2 and muscle weight (*r* = −0.168, *P* = 0.19) (Table 4). These results suggest that HOMA-IR had an intervention effect of serum LECT2 on muscle weight,

## Discussion

It is very important to verify the impact of an HF diet on aging-related diseases in a way that would reflect the dietary patterns of modern society [31]. Clinical and experimental studies have clearly documented that aging and obesity-related diseases are risk factors for physical disability accompanying muscle atrophy, but the effect of HF diet on aging-related muscle atrophy is not well documented. Our experiments suggest that skeletal muscles were more susceptible to aging in SAMP8 mice than SAMR1 mice and that the loss of muscle mass with aging was exacerbated in SAMP8 mice fed an HF diet. These findings suggest that SAMP8 mice are an appropriate model for muscle atrophy. In these mice, GTEs ameliorated the loss of muscle mass and improved insulin sensitivity in skeletal muscle.

Guo et al. reported that muscle weight of SAMP8 mice peaked at 7 months of age and significantly declined by 12% at 10 months, which state was considered under muscle atrophy [12]. In our experiment, the muscle weight declined by 15.3% in mice fed an HF diet from 2 to 6 months of age, whereas GTEs increased muscle weight by 22.1% in the HFGT group in comparison with the HF group. The magnitude of group differences in muscle weight was similar to that in the report by Guo et al. [12]. Therefore, the decline in muscle weight might reflect muscle atrophy in aging SAMP8 mice fed an HF diet and GTEs might alleviate muscle atrophy.

Several factors are likely to contribute to the loss of muscle weight, including poor nutrition, muscle disuse, reduced protein synthesis, and insulin resistance [18, 32, 33]. We did not measure the daily amount of activity, but food intake did not differ between SAMR1 and SAMP8 mice. In disused muscles, suppression of insulin signaling reduces protein synthesis and simultaneously activates protein degradation [16, 34]. Accordingly, we focused on insulin sensitivity as an index of protein synthesis, and on protein degradation, as mechanisms of changes in skeletal muscle mass. We did not directly measure protein synthesis, but it may have been reduced in HF diet–fed mice, because insulin signaling in the HF group tended to be decreased as evidenced by decreased phosphorylation levels of Akt and S6K. These kinases act upstream of protein synthesis [19]. In addition, HOMA-IR, an index of insulin resistance, was negatively correlated with muscle weight. On the other hand, protein degradation did not differ among groups, as evidenced by the similar levels of MuRF1 and Fbx32, total ubiquitination, and phosphorylation of FoxO1 and FoxO4 in skeletal muscle (data not shown). Consequently, we assume that insulin sensitivity is one of the important factors in skeletal muscle mass changes.

Little has been done to clarify the fundamental mechanisms of the induction of insulin resistance in skeletal muscle, especially the contribution of communication between organs. One of the mechanisms of such communication is the regulation of insulin sensitivity by serum LECT2 [28]. Lan et al. [28] reported that serum LECT2 concentrations are correlated positively with HOMA-IR and negatively with insulin sensitivity in humans. *Lect2* deletion attenuates muscle insulin resistance in dietary obese mice, and *Lect2* transfection in myocytes decreases insulin signaling [28]. In our experiments, serum LECT2 concentrations in SAMP8 mice were positively correlated with HOMA-IR. In HF-fed mice, serum LECT2 concentrations were elevated and insulin signaling in skeletal muscle tended to be reduced. While we have no direct evidence that serum LECT2 concentrations are associated with muscle weight, HOMA-IR was correlated with muscle weight and had an intervention effect of serum LECT2 on muscle weight.

Our findings indicate that GTEs improved insulin signaling in skeletal muscle and reduced serum LECT2 level. It needs to be investigated to what extent serum LECT2 is involved in the effect of GTEs on insulin sensitivity in skeletal muscle and how GTEs reduce serum LECT2 concentrations. Adenosine-monophosphate-activated protein kinase (AMPK) might be associated with the effects of GTEs. AMPK reduces LECT2 production in hepatocytes [28]. Supplementation with GTEs for 16 weeks activates AMPK in the livers of HF-fed mice [35], and AMPK is activated after single oral administration of green tea in mice [36]. Although we did not examine AMPK activity, GTEs might reduce serum LECT2 concentrations by inducing unknown metabolic signaling pathways in the liver.

To help prevent muscle atrophy in elderly people, nutritional and resistance–training intervention studies have been conducted worldwide [7, 37–40]. These studies suggest that nutrient supplementation and resistance training increase lean body mass, but their effects in the elderly are limited. Insulin action in skeletal muscle is essential for protein synthesis during the rest phase [41]. However, it has been reported that a decrease in insulin action in the muscle reduces protein synthesis in the elderly, even when they take amino acid or glucose supplements and receive insulin infusions [42, 43]. Suppression of hepatokines improves not only insulin sensitivity in skeletal muscle but also the effects of resistance training [28, 44, 45]. Although our study provided no direct evidence for the influence of serum LECT2 concentrations on muscle weight, HOMA-IR had an intervention effect of serum LECT2 concentrations on muscle weight. For this reason, the reduction in the levels of hepatokines such as serum LECT2 might be important for improving insulin sensitivity in skeletal muscle and is possibly associated with muscle weight via insulin resistance.

## Conclusions

Our experiments showed that excessive intake of fats exacerbated aging-related loss of muscle weight accompanied by insulin resistance, whereas the intake of GTEs ameliorated insulin signaling in skeletal muscle and alleviated muscle weight loss in SAMP8 mice. GTEs also reduced serum LECT2 concentrations, which were correlated with insulin resistance. Although we have no direct evidence for the effect of serum LECT2 on muscle weight, insulin resistance assessed as HOMA-IR had an intervention effect of serum LECT2 on muscle weight. Future studies will be needed to reveal the mechanisms of the effects of GTEs on muscle weight and LECT2 levels.

## Supporting information

S1 Appendix Phosphorylation of kinases involved in insulin signaling in skeletal muscle of adult (6M) SAMP8 mice. Western blot images were shown for p-Akt (A), Akt (B), p-S6K (C), and S6K (D). Precision Plus Protein^™^ Dual Color Standards (Bio-Rad Laboratories, Inc., Hercules, CA) were used for molecular markers.
(TIF)
