# Supplementary figures and images for: Green tea extracts ameliorate high-fat diet–induced muscle atrophy in senescence-accelerated mouse prone-8 mice
Source: PLoS One. 2018 Apr 9;13(4):e0195753. doi: 10.1371/journal.pone.0195753 (PMC5891070; doi:10.1371/journal.pone.0195753)

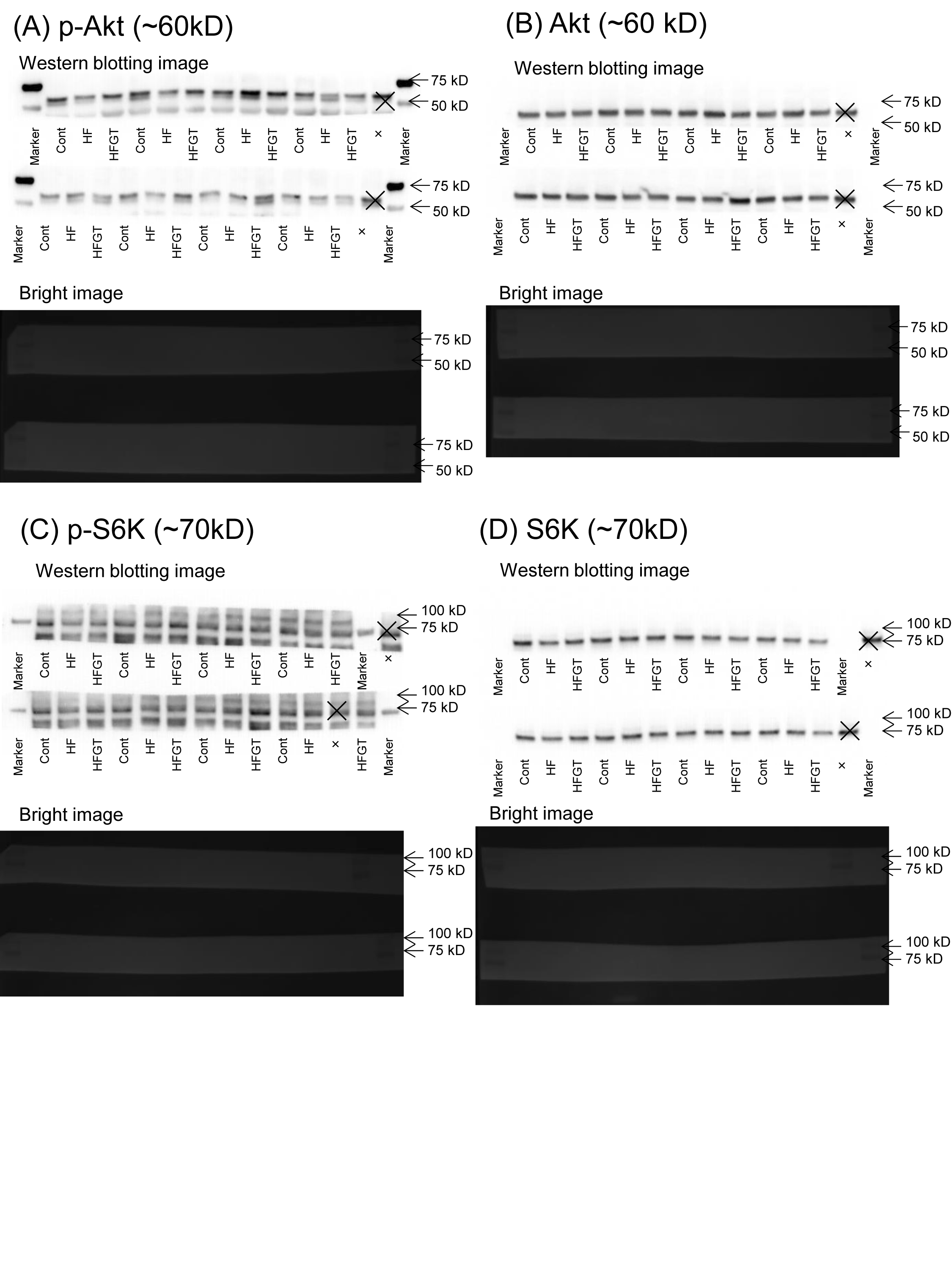

Supplement: S1 Appendix — Western blot images were shown for p-Akt (A), Akt (B), p-S6K (C), and S6K (D). Precision Plus Protein™ Dual Color Standards (Bio-Rad Laboratories, Inc., Hercules, CA) were used for molecular markers. (TIF) [file pone.0195753.s001.tif]
